# Multiple Sclerosis and Serotonin: Potential Therapeutic Applications

**DOI:** 10.7759/cureus.11293

**Published:** 2020-11-02

**Authors:** Aleyda M San Hernandez, Chetana Singh, Danel J Valero, Javariya Nisar, Jose I Trujillo Ramirez, Karisma K Kothari, Sasank Isola, Domonick K Gordon

**Affiliations:** 1 Internal Medicine, California Institute of Behavioral Neurosciences & Psychology, Fairfield, USA; 2 Primary Care, California Institute of Behavioral Neurosciences & Psychology, Fairfield, USA; 3 Anesthesia, California Institute of Behavioral Neurosciences & Psychology, Fairfield, USA; 4 Internal Medicine, Scarborough General Hospital, Scarborough, TTO

**Keywords:** multiple sclerosis and serotonin, serotonin, multiple sclerosis, chemistry of multiple sclerosis

## Abstract

Multiple sclerosis (MS) is a neurodegenerative disease with a complex autoimmune component, and it has a high prevalence among middle-aged females. The manifestations of the disease range from episodic somatosensory dysfunction to progressive and permanent central nervous system (CNS) damage. Due to a high prevalence of psychiatric comorbidities and proven abnormalities in serotonin (5-HT) levels among MS patients, they are usually on drugs that modify the serotonergic system. Through a comprehensive literature review of studies published in the last 10 years related to 5-HT in MS and its therapeutic applications, we aimed to elucidate the mechanism behind the neurotransmitter (NT) levels’ abnormalities. Most importantly, we endeavored to gather the most up-to-date information about the full therapeutic potential of agents acting on this system. We discovered that multiple processes cause low levels of 5-HT in MS patients. The varying levels of the availability of the 5-HT transporter (SERT) in the CNS decreasing overall tryptophan (TRP) levels, and diversion of the amino acid away from its synthetic pathway constitute some of those. Studies in animals have shown that 5-HT levels’ elevations could cause immune-modulating effects and could probably slow down the disease progression rate. Human studies have shown a more diverse and complex response. Promising results have been obtained in the last 10 years regarding 5-HT’s immune-modulatory role in MS patients and its therapeutic applications. Human studies with a larger population and feasible designs are still needed to fully ascertain the effects of serotonin on the immune system and disease progression in patients with MS.

## Introduction and background

It is estimated that there are more than two million people suffering from multiple sclerosis (MS) globally [1]. According to recent studies conducted by the National Multiple Sclerosis Society, there are nearly one million MS patients in the United States (US) alone.

MS is a chronic neuroinflammatory disease that causes significant distress and progressive disability in middle-aged adults by impairing motor, autonomic, and cognitive functions [2]. The condition has a higher prevalence in females who are between 20-50 years of age. Its pathophysiology is based on vascular, inflammatory, and autoimmune mechanisms that closely interact, leading to oligodendrocyte dysfunction and demyelination of neurons in the central nervous system (CNS). At the vascular level, there is usually a disruption of the blood-brain barrier (BBB), which leads to the infiltration of CNS by immune cells. Once in the CNS, these immune cells induce an inflammatory reaction wherein toxic metabolites are released, thereby destroying nearby cells, especially neurons and oligodendrocytes. Through a series of chemical mediators-receptor interactions, the immune damage is further aggravated, contributing to cumulative irreversible changes seen in later stages in the disease process [3]. Based on this rationale, MS is usually classified as relapsing-remitting MS (RRMS), associated with the local inflammation seen in more than half of the patients at the time of diagnosis, or secondary progressive MS (SPMS), which usually follows a more severe course correlated to cumulative neurodegeneration [4]. The current treatment modality for MS focuses mainly on symptomatic relief during flare-up episodes with minimal effects on disease progression. Corticosteroids are the primary drugs used during acute inflammation episodes. Immune-modulating therapy encompasses drugs like glatiramer acetate and interferon-beta.

Serotonin (5-HT) is an endogenous neurotransmitter (NT) in the human body, synthesized from the essential amino acid (AA) tryptophan (TRP). TRP is converted into 5-HT via a group of chemical reactions in which the enzyme tryptophan hydroxylase (TPH) plays an important role [5]. 5-HT has various receptor types and locations, allowing for its diverse yet specific effects on structures throughout the body. Some of the most studied sites of action of 5-HT are the gastrointestinal system, cerebral cortex, hypothalamus, and limbic system. This NT modulates cognition, mood, sleep, hunger, sexual behavior, and multiple gastrointestinal functions [6].

Psychiatric comorbidities are prevalent in MS patients, especially those caused by abnormal 5-HT levels [7]. Studies have shown that serotonergic drugs in mammals with experimental autoimmune encephalopathy (EAE), an animal form of MS, causes not only symptomatic relief but also some level of neuroprotection [8]. The causes and effects of abnormal 5-HT levels in MS patients are still not fully understood. The efficacy of treatments that slow down the disease progression in humans through serotonergic pathways is still being debated.

Through a comprehensive literature review, this study attempts to elucidate the mechanisms behind the abnormalities in 5-HT levels in MS patients and their effect on the disease progression. As a secondary objective, we will gather and discuss the most recent evidence supporting 5-HT-modifying drugs as a starting point for future studies.

## Review

We conducted a literature review using PubMed, Medline, and PubMed Central databases. We extracted the data using keywords like "Multiple sclerosis and serotonin," "Serotonin," "Multiple Sclerosis" and "chemistry of Multiple Sclerosis". Table 1 shows the number of article yields by each keyword. We screened the studies by reading the abstracts, and according to their significance to our topic; then the full reports were read and were selected based on our inclusion-exclusion criteria. Additional relevant articles were found using the references in the identified studies. Only those articles related to our topic and published within the last 10 years (2010-2020) were included. Studies conducted within and outside the US and with multiple designs were reviewed.

**Table 1:** Article yield by keyword

Keyword	Number of articles	Database
Multiple sclerosis	43,747	PubMed
Serotonin levels	9,720	PubMed
Multiple sclerosis and serotonin	129	PubMed
Chemistry of multiple sclerosis	4,401	PubMed

Psychiatric pathologies like major depressive disorder (MDD) have a high prevalence in MS patients. In the last few decades, multiple studies have tried to comprehend the mechanism behind this phenomenon, focusing primarily on 5-HT levels and the reason behind its abnormalities. Drugs that modify the serotonergic system have been extensively used in this population and, more than providing symptomatic relief, these agents have been shown to alter the immune system and disease progression. This opens a window for alternative treatments for MS patients, a disease that has been managed mostly in a symptomatic manner so far.

Serotonin levels in MS patients

Studies in MS patients have shown that serum and platelet 5-HT levels are diminished in this population [9,10]. To elucidate this phenomenon, 5-HT transporters (SERT) availability has been studied in this population. In 2014, a study involving 45 individuals concluded that, compared with healthy volunteers, patients with RRMS had decreased transporter availability in the limbic system. Patients with PPMS had increased SERT expression in the prefrontal areas [11]. Even though a small sample size was used in this study, it opened the doors for future investigations. Four years later, with a sample size of 200, a research group tried to elucidate SERT gene polymorphism's role in regulating serotonin levels. No difference was found between MS patients and healthy volunteers, and this suggested that perhaps other mechanisms are involved in this process [2].

**Figure 1:**
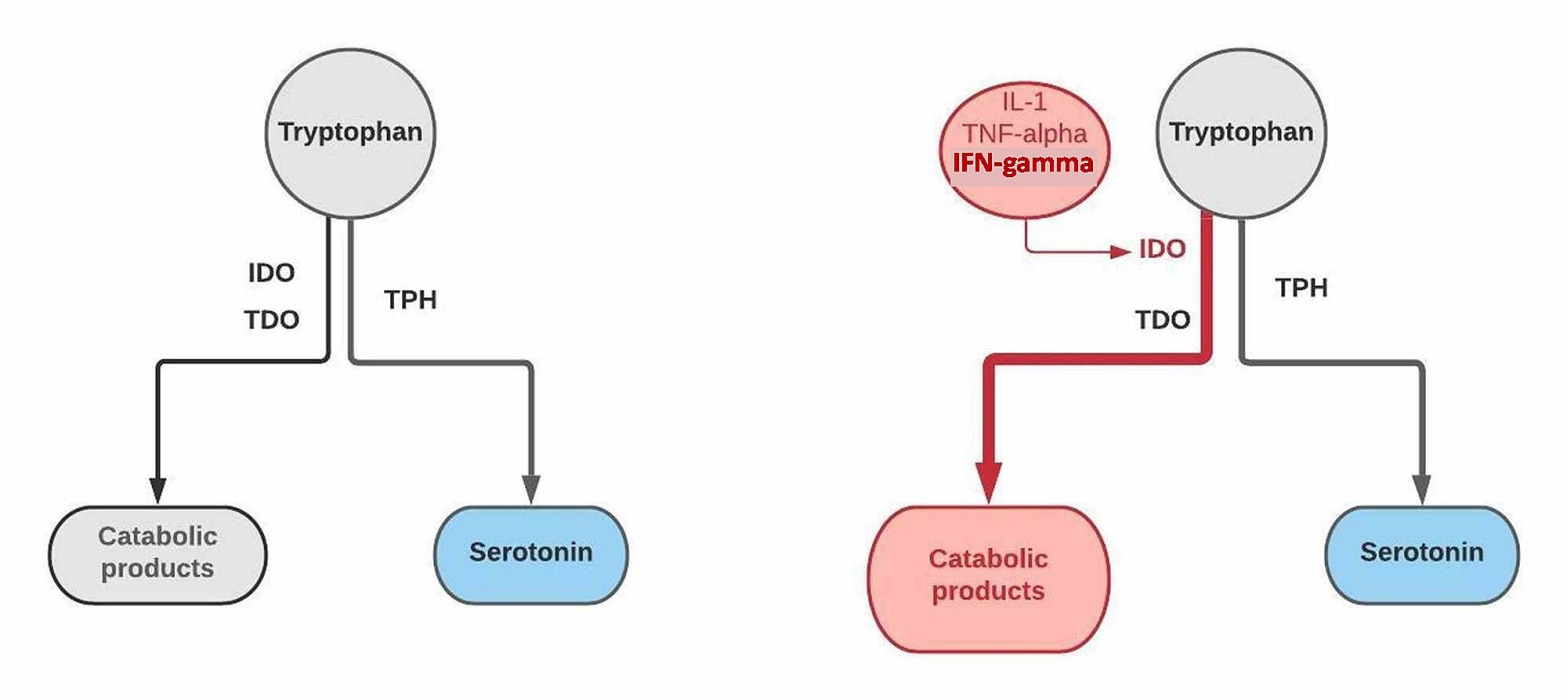
Tryptophan metabolism in MS patients The figure highlights one of the plausible mechanisms causing decreased levels of serotonin in MS patients. The diagram on the left represents normal tryptophan metabolism. A synthetic pathway using tryptophan hydroxylase (TPH) allows serotonin formation, while enzymes IDO and TDO degrade tryptophan into various products. The diagram on the right highlights the diversion of tryptophan into this catabolic pathway through induction of the enzyme IDO by inflammatory cytokines like IL-1, TNF-α, and IFN-γ present in high levels during inflammatory periods in MS patients MS: multiple sclerosis; IDO: indoleamine 2,3-dioxygenase; TDO: tryptophan dioxygenase; IL-1: interleukin-1; TNF-α: tumor necrosis factor alpha; IFN-γ: interferon gamma; TPH: tryptophan hydroxylase

A plausible explanation could lie in the synthesis of the NT. 5-HT is synthesized from the essential AA called TRP, which can be metabolized through two main pathways: a synthetic pathway, and a catabolic pathway - the kynurenine pathway (KP) [12]. The enzyme TPH plays a critical role in the synthetic path that gives rise to 5-HT, melatonin, and other products through a series of chemical reactions. The KP, a catabolic route, involves enzymes like indoleamine 2,3-dioxygenase (IDO) and tryptophan dioxygenase (TDO) [13]. Elevated activity of the KP has been reported in patients with MS, especially in those with the relapsing-remitting subtype [2], driving TRP into its catabolic pathway and away from the synthesis of the NT. This diversion is accomplished primarily through the induction of the enzyme IDO by cytokines like interferon-gamma (IFN-γ), interleukin-1 (IL-1), and tumor necrosis factor-alpha (TNF-α) released in high titers in the inflammatory foci [14]. Figure 1 below summarizes the two major metabolic pathways of TRP and its modification in MS patients.

Studies have shown an increased risk of developing the disease in high latitudes; it has also demonstrated a decrease in TRP consumption in such areas [15]. Hence, the low availability of the AA could explain the decreased levels of the NT. Finally, studies in animals and humans have shown damage to the neurons responsible for the synthesis of 5-HT in the bulbospinal area [16]. Overall, a variety of processes driving the NT modulation have been described. Future investigations, with bigger sample sizes, will tell us the exact mechanisms behind the 5-HT levels' abnormalities in MS patients.

Serotonin's Effects on MS Patients

Among the different inflammatory cells present in MS lesions, a vital role is played by macrophages. Two important subtypes are commonly identified: M1 and M2. M1 is a pro-inflammatory subtype activated by substances like IFN-γ and lipopolysaccharide (LPS), and it secretes toxic metabolites that further increase the inflammatory reaction. Simultaneously, M2 is a regulatory subtype activated by interleukin-4 (IL-4) among other cytokines, and it secretes anti-inflammatory mediators that prevent the foci from further propagation. As the disease progresses, the M1 macrophage becomes more prevalent than M2 [17]. Different 5-HT receptors have been identified on macrophages, specifically 5-HT_2B_ and 5-HT_7_ receptor activation, which increase the proportion of macrophage polarization towards the M2 subtype [15].

Different T cell subsets are present in the disease foci. The Th1 population, through the production of IFN-γ, and the Th17 population, via interleukin-17 (IL-17) and interleukin-22 (IL-22) synthesis, predominate during acute episodes and contribute to the oligodendrocytes death and neuron demyelination. Meanwhile, CD4+ T cells secrete a group of regulatory cytokines like interleukin-10 (IL-10), to decrease the inflammation [18]. Due to the variety of 5-HT receptors on the lymphocytes, the NT exerts a wide range of effects. Through 5-HT_1A_ receptor activation, in CD4+ cells, 5-HT increases the production of IL-10. At the opposite end, the activation of the 5-HT_3_ receptor appears to activate T cells and produce inflammatory mediators like interleukin-6 (IL-6) and IL-17 [15]. An in vitro study on MS patient's peripheral blood mononuclear cells (PBMCs), conducted in 2016, showed increased activity of TH1 and Th17 subsets on the production of IL-17 and IFN-γ in relapsing patients and a decrease in IL-17 production after the addition of 5-HT [19]. In 2018, a similar study on T cells of MS patients showed that exogenous 5-HT decreased the synthesis of IL-17, IL-22, and IFN-γ in the Th1 and Th17 cell populations. The same study also reported a decrease in T cell proliferation and the synthesis of TNF-α after the 5-HT infusion, and as previously mentioned, they found an increase in regulatory cytokines release [18]. Even though both studies centered only on patients with RRMS and could not show any clinical improvement, their findings still provide a closer look at the possible neuroprotective function of 5-HT in MS patients.

**Figure 2:**
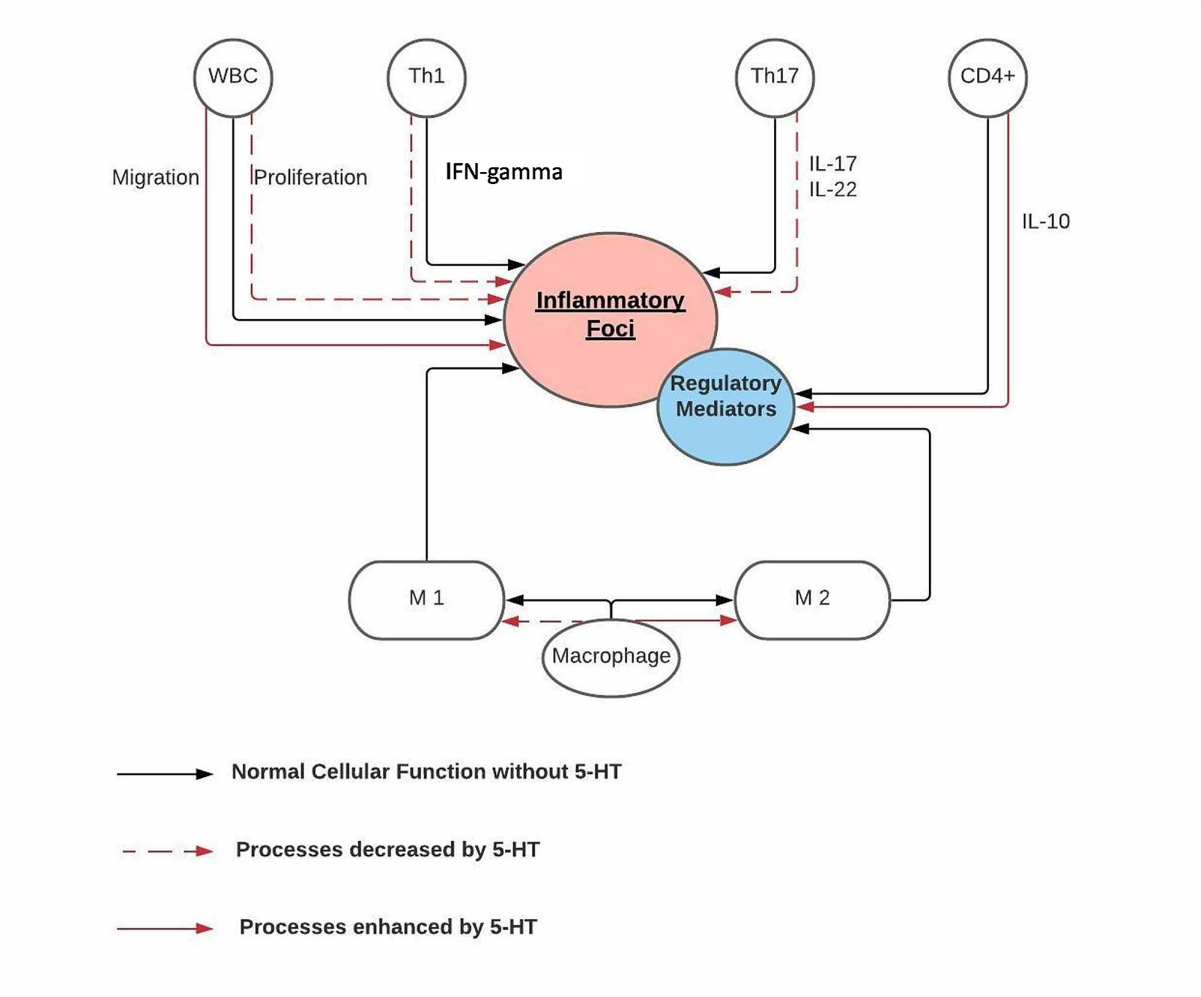
Serotonin's effects on the immune system The figure summarizes the findings of previous studies about the effects of serotonin on the immune system. Overall, 5-HT decreases the proliferation of WBC, suppresses the synthesis of inflammatory cytokines like IFN-γ, IL-17, and IL-22 by TH1 and Th2 cells, respectively. The NT also enhances the synthesis of IL-10, a regulatory cytokine, from CD4+ cells and acts on macrophages, enhancing its polarization into the M2 subset, a population that secretes regulatory mediators to control inflammation. Finally, the addition of 5-HT to WBCs increases their migration through the BBB into the inflammatory foci 5-HT: serotonin; IFN-γ: interferon gamma; IL: interleukin; WBC: white blood cells; NT: neurotransmitter

A critical step in the disease's pathophysiology is the migration of the white blood cells (WBCs) and other immune cells from the periphery through the BBB into the CNS's inflammation foci. A study conducted in 2016 showed an increased activation and movement of leukocytes through an endothelial cell layer after the infusion of 5-HT [3]. Even though the effect of 5-HT was seen in healthy volunteer cells in this study, it is still a valid variation from the discussed neuroprotective effect of 5-HT and opens a window for future investigations. Figure 2 below summarizes the significant effects of 5-HT on the immune cells in MS patients.

Oligodendrocytes are the cells responsible for the myelination of neurons in the CNS and, along with neurons, are the primary targets for the immune attack on MS patients. Two different 5-HT receptors have been identified on oligodendrocytes: 5-HT_1A_ and 5-HT_2A_ [20]. The 5HT_1A_ receptors have been identified on immune cells like monocytes and dendritic cells, and their activation has been related to the anti-inflammatory effects [21].

From a more clinical perspective, multiple studies have found associations between increased 5-HT levels and improvement in MS patients' health status. A study conducted in 2016 showed that physical activity decreases the fatigue severity scale (FSS), and the study associated this improvement with an elevation of the 5-HT levels present in the same group [22]. Likewise, a randomized controlled trial (RCT) in 2019 showed an increase in 5-HT levels with aerobic exercise, and it associated this phenomenon with the improvement in the participants' sleep quality [23]. Although both studies had a small sample size, the results are promising for future therapies on targeting the serotonergic system to improve the overall health of MS patients and their quality of life.

Serotonergic System-Modulating Drugs

Serotonin reuptake inhibitors (SSRIs) are usually prescribed to MS patients for the treatment of psychiatric comorbidities. Increasing interest over its effect on disease progression and immune modulation has been observed during the last few years. A study conducted in 2012 showed that the use of SSRI in animals with EAE prophylactically increased the remission periods [24]. Later studies have shown that the use of SSRIs after the onset of the symptoms can slow down the rate of progression of the disease [25], decrease clinical scores as well as symptomatic frequency and intensity [8,26]. These clinical improvements can be explained by the immunomodulatory capacity of the drugs. SSRIs in EAE animals suppress the synthesis of inflammatory cytokines like IFN-γ, TNF-α, IL-6, IL-10, and IL-2 [25,26]. Simultaneously, the antidepressant upregulates the synthesis of anti-inflammatory mediators like IL-4 [8], and it can induce T cell apoptosis [26].

A comprehensive study conducted in 2017 showed that fluvoxamine could act on neural stem cells. At different concentrations, fluvoxamine was able to augment the viability of these cells and their proliferation capacity, and also their ability to transform into oligodendrocytes [8]. Another critical effect found on SSRI-treated animals with EAE was a decrease in the inflammatory lesions and the degree of demyelination in the CNS [8,24]. Overall, studies have shown that SSRIs have a neuroprotective and anti-inflammatory role in EAE animals. However, It still remains a challenge to extrapolate these results into humans, and there is still uncertainty about the specific dosage of the drugs to be administered since, in most of these studies, higher dosages of SSRIs increased the number of animal deaths. Table 2 below highlights the essential points put forward by the previously discussed studies.

**Table 2:** Comparison of different studies that evaluate the different effects of SSRIs on EAE animals YOP: year of publication; SSRI: selective serotonin reuptake inhibitor; EAE: experimental autoimmune encephalopathy; IFN-γ: interferon gamma; TNF: tumor necrosis factor; IL: interleukin

Title	Author	YOP	Purpose of the study	Conclusions
The immunomodulatory effect of the antidepressant sertraline in an experimental autoimmune encephalomyelitis mouse model of multiple sclerosis	Taler et al. [25]	2011	Evaluate the immunosuppressive capacity of sertraline in EAE animals	Sertraline was able to slow the progression of the disease and ameliorate some of the symptoms and decrease the synthesis of multiple cytokines like IFN, TNF, and IL-2 after the infusion of the drug
Fluoxetine promotes remission in acute experimental autoimmune encephalomyelitis in rats	Yuan et al. [24]	2012	Determine the effects of fluoxetine used prophylactically in EAE animals	Fluoxetine effects: suppression of IFN-γ synthesis; decreased the number of inflammatory lesions on the CNS and the demyelination degree
Amelioration of ongoing experimental autoimmune encephalomyelitis with fluoxetine	Bhat et al. [26]	2017	Determine the action of fluoxetine as in immune modulator	Fluoxetine was able to suppress the synthesis of inflammatory cytokines (TNF-α, IL-6, and IL-10) and decrease the intensity and frequency of symptoms; induced cell death on T lymphocytes
Fluvoxamine stimulates oligodendrogenesis of cultured neural stem cells and attenuates inflammation and demyelination in an animal model of multiple sclerosis	Ghareghani et al. [8]	2017	Evaluation of the effects of fluvoxamine in EAE animals	Fluvoxamine could act on neural stem cells: augmented the viability of these cells and their proliferation capacity; induced conversion into oligodendrocytes; decreased clinical scores, inflammatory foci, and demyelinated areas after the SSRI treatment; decreased IFN-γ synthesis; increased IL-4

Phenelzine is a commonly used monoamine oxidase inhibitor (MAOI), a class of antidepressants that, by enzyme inhibition, increases neurotransmitters like 5-HT, melatonin, and norepinephrine, among others. Studies using this drug, both prophylactic and after the induction of the disease, have shown clinical improvement in EAE animals but failed to demonstrate any reduction in the extent of the inflammatory or demyelinating lesions in the CNS [27,28]. In 2015, a research team studied for the first time the effects of aryl piperazine D2/5-HT_1A_ ligands in rats with EAE. It demonstrated not only symptomatic improvement and a delay in the disease's progression, but also a decreased cell death rate with the protection of oligodendrocytes and neurons from T lymphocytes [29]. Even though the above studies have shown amelioration of EAE in animals and, in some cases, protection against the immune attack, it is essential to consider that both agents act on more than just the serotonergic system. The concurrent modulation of various NTs, along with 5-HT, opens up a broader avenue for treating the disease. Finally, a study conducted in 2013 showed that serotonin receptor antagonists exert positive impacts on EAE animals, which contrasted the previously discussed effects of serotonergic-modifying drugs. Decreased infiltration of the CNS by immune cells, demyelination, and synthesis of inflammatory cytokines resulted in animals being successfully treated and symptomatic improvement [30].

In 2013, an RCT was conducted on 42 patients with progressive MS to gain insight into the effects of fluoxetine in this population. After two years of follow-up, no significant difference in the disease progression, either clinical or radiological, was found between the fluoxetine-treated patients and the placebo group [31]. Six years later, a research group followed 137 patients with progressive MS randomized into fluoxetine-treated and placebo group for a period of 108 weeks to elucidate the neuroprotective ability of fluoxetine. Again, the study failed to demonstrate any significant difference between the two groups [32]. A more recent study, using the same SSRI in patients with secondary progressive MS for 96 weeks, also failed to demonstrate any difference between the rate of brain volume loss among these patients and that in the control group [33]. In 2016, a case-control study with a sample size of 3,920 MS patients attempted to evaluate the association between SSRI exposure and disability progression rate. No delay in the disability rate was found in patients exposed to SSRI; instead, an increasing level of disability was found in patients highly exposed to SSRI who did not develop secondary progressive disease [34]. Even though most studies in humans have failed to demonstrate the neuroprotective effects of SSRI in MS patients, it is still early days in the investigation of this topic. Small sample sizes, confounding bias, and unexpected findings are some of the difficulties faced by researchers in the field. Table 3 highlights the important points arrived at by the analyzed studies.

**Table 3:** Comparison of studies conducted in humans with MS YOP: year of publication; MS: multiple sclerosis; SSRI: selective serotonin reuptake inhibitor; RCT: randomized controlled trial

Title of the study	Author	YOP	Study design	Number of patients	Purpose of the study	Conclusions
The effect of fluoxetine on progression in progressive multiple sclerosis: a double-blind, randomized, placebo-controlled trial	Mostert et al. [31]	2013	RCT	42	Evaluate the ability of fluoxetine to modify clinical and radiologic parameters in patients with progressive MS	No significant clinical or radiologic improvement was detected in the fluoxetine-treated group in comparison with the control
Association between the use of selective serotonin reuptake inhibitors and multiple sclerosis disability progression	Zhang et al. [34]	2016	Case-control	3,920	Determine if the exposure to SSRI is associated with a slow rate of disability in MS patients	No association was found between the use of SSRI and a slower disability accumulation rate
Fluoxetine in progressive multiple sclerosis: the FLUOX-PMS trial	Cambron et al. [32]	2019	RCT	136	Determine if treatment with fluoxetine could slow the progression of progressive MS	No significant difference in clinical scores or imaging findings were found between the two compared groups
Amiloride, fluoxetine or riluzole to reduce brain volume loss in secondary progressive multiple sclerosis: the MS-SMART four-arm RCT	De Angelis et al. [33]	2020	RCT	440 was the target sample size, only 111 were taking fluoxetine	Determine whether fluoxetine could decrease the brain atrophy rate in progressive MS patients	No difference in brain loss percentage between the patients taking fluoxetine and the controls

Even though we collected the information from multiple study design articles, from research conducted within and outside the US, and did not exclude articles based on quality assessment tool scores, we only included articles from the last five years relevant to our topic. Using this method, we attempted to gather the most significant amount of up-to-date information about the subject and highlight the importance of further investigations with bigger sample sizes of humans and affordable designs, which will let us determine the full therapeutic effects of serotonin-modulating drugs.

## Conclusions

Abnormal levels of serotonin and psychiatric comorbidities involving this NT are highly prevalent in MS patients. However, it is still a question of the mechanism behind this phenomenon, and most importantly, what therapeutic implications could have a modification in the NT processing. A majority of studies showed decreased levels of serotonin in MS patients and an improvement in clinical scores after the use of drugs that increase its levels. In vitro and animal studies showed that increased serotonin levels have an anti-inflammatory and immunosuppressive action that may decrease the disease’s progression. There is still not enough evidence to confirm these findings in humans. Further research with affordable study designs and bigger sample sizes is recommended to elucidate the full potential of serotonergic drugs in MS patients.
